# Supplementary material for: Innovation and cumulative culture through tweaks and leaps in online programming contests
Source: Nat Commun. 2018 Jun 13;9:2321. doi: 10.1038/s41467-018-04494-0 (PMC5998038; doi:10.1038/s41467-018-04494-0)
Supplement: Supplementary file 1 — Supplementary Information [file 41467_2018_4494_MOESM1_ESM.pdf]

SUPPLEMENTARY INFORMATION

Innovation and Cumulative Culture through 'Tweaks' and 'Leaps' in Online Programming Contests

Miu et al.

Contents:

Supplementary Figures 1-8
Supplementary Methods

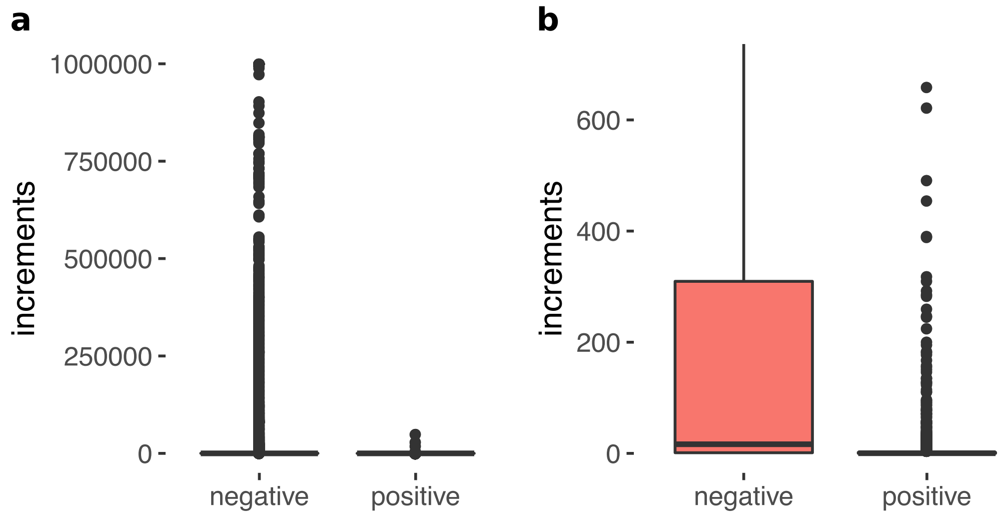

Supplementary Figure 1

Boxplots of the distribution of negative/positive incremental improvements in all the contests (a), and zoomed in on the data below the third quantile (b). Incremental improvements are calculated as the difference in score between each entry and the current best scoring entry so far – positive increments represent entries that improve the overall score.

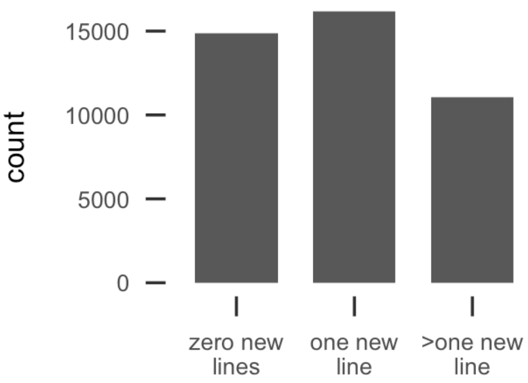

Supplementary Figure 2

Total number of entries introducing zero, one, or more than one new lines of code, across all contests. Most entries introduce new information.

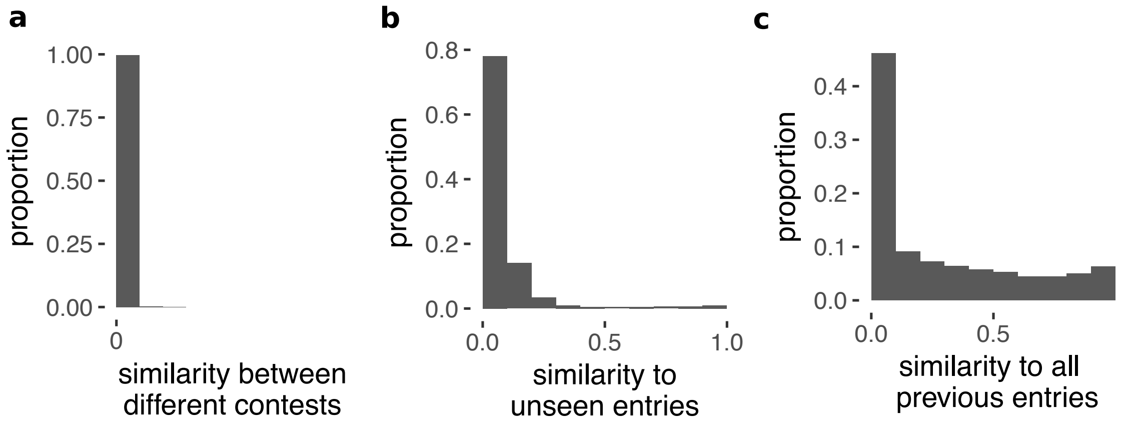

Supplementary Figure 3

(a) Baseline similarity between all the entries in two different randomly chosen contests; (b) baseline similarity between all the entries in the 'darkness' and 'twilight' conditions in all contests, in which participants only had access to information regarding their own entries; (c) baseline similarity between all entries and all the previous entries submitted in all contests. None of the baseline similarity distributions displays patterns similar to the distribution of similarities to the current leader.

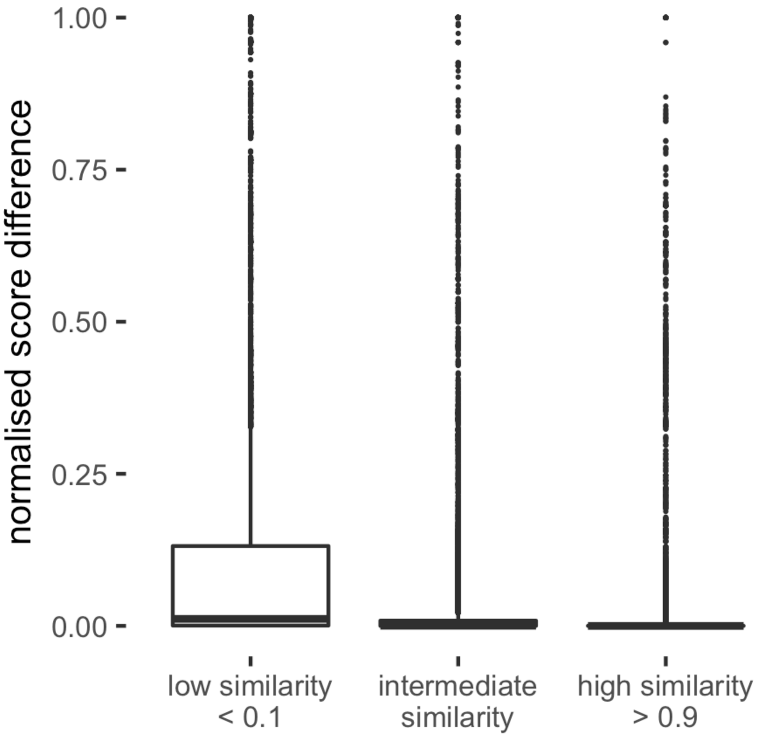

Supplementary Figure 4

Normalised score difference between each entry and the current leader, as a function of code similarity to the current leader. The more similar an entry to the current leader in terms of code, the smaller the difference in score.

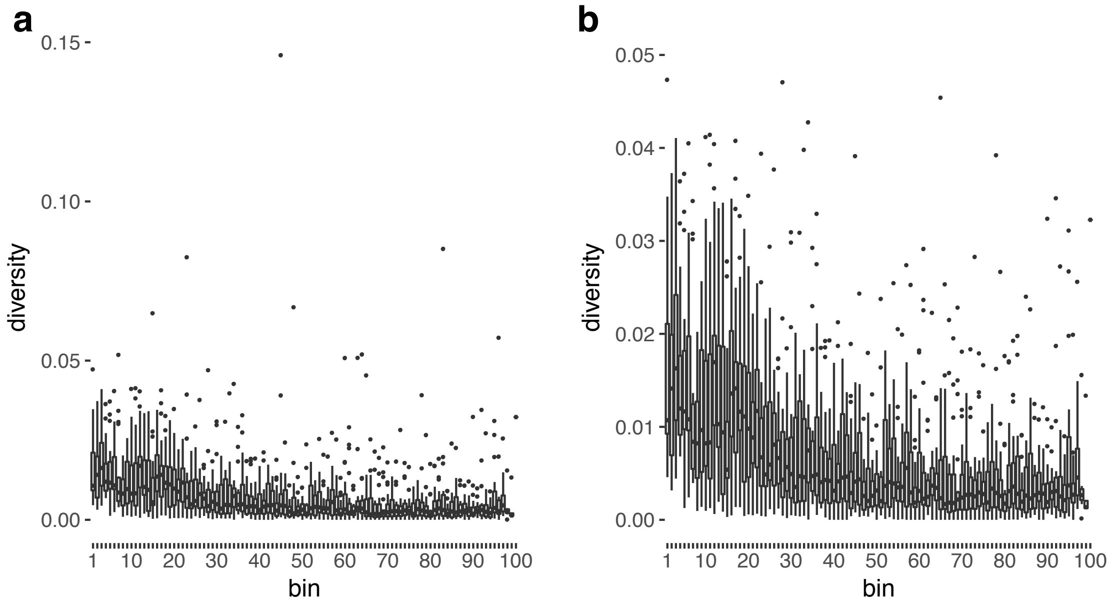

Supplementary Figure 5

Distribution of cultural diversity values across all contests for each 1% contest progress bin (a), and adjusted to exclude outliers in (b). For ease of visualization, the cultural diversity values were ordered chronologically and grouped into bins covering each 1% of the data. Thus, increasing bin values indicate time progression – for each bin we plotted a boxplot of the diversity distribution in that time interval.

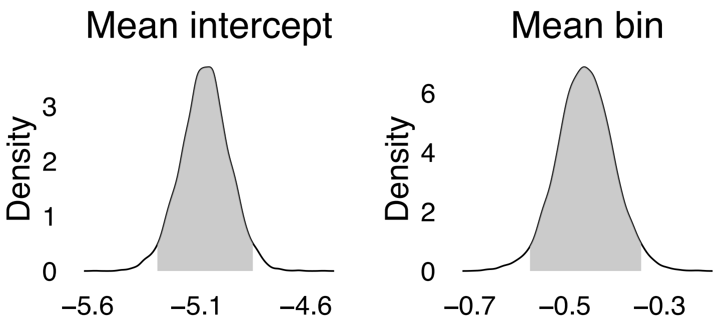

Supplementary Figure 6

Posterior distributions of parameter estimates for intercept and standardised time bin from generalised linear mixed-model predictions of cultural diversity over time. The shaded area indicates the 95% credible interval of the posterior estimates per contest.

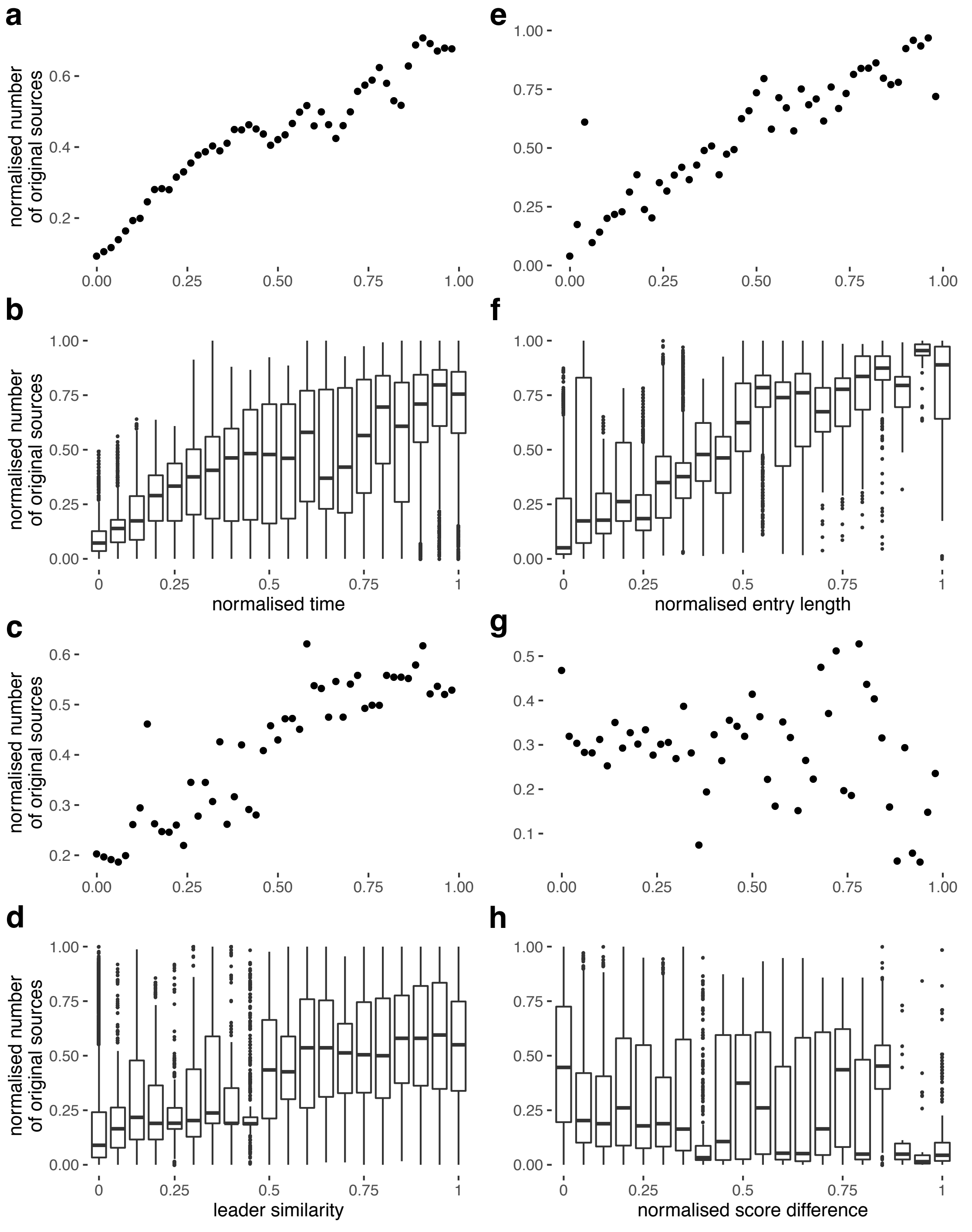

Supplementary Figure 7

Normalised number of original sources for all entries as a function of time (a, b), similarity to the current leader (c, d), entry length (e, f) and score difference to the current leader (g, h). For ease of visualization, each of the four variables has been split into bins, and for each bin we have calculated the average number of original sources (a, c, e, g – over 50 bins), and the distribution of the number of original sources in that bin are plotted below (b, d, f, h – over 20 bins).

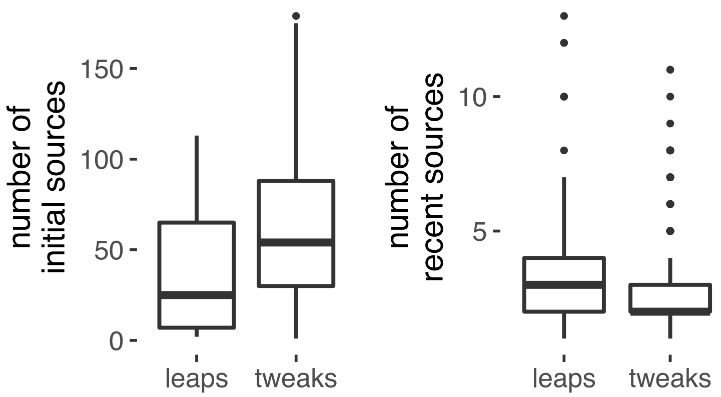

Supplementary Figure 8

Number of initial and recent sources for successful tweaks (i.e. leaders with a similarity > 0.9 to the previous leader) and leaps (i.e. leaders with a similarity < 0.1 to the previous leader).

Supplementary Methods

Quantifying tweaks and leaps

The distribution of similarities between entries and the current leading entry splits the entries into common tweaks of the current leader, and a rare number of leaps that diverge drastically from the leading solution. There is no clear objective threshold that separates entries into tweaks and leaps, but given that, in the 'darkness' and 'twilight' conditions in which players can only receive information regarding their own entries, the baseline similarity between entries within the same contest averages approximately 0.1, we chose to classify entries as 'leaps' if their similarity to the current leader is under 0.1, and as 'tweaks' when the similarity to the current leader in over 0.9. According to this split, the 'leaps' amount to 21.87% of all 'daylight' entries in all contests, while 'tweaks' amount to 52%, thus resulting into a proportion of tweaks over leaps of 2.38. Visually inspecting the distribution of similarities to the current leader (Fig. 2b) suggests a large number of entries cluster over the value of 0.7. Therefore, if we choose to classify an entry as a 'leap' if it has a leader similarity lower than 0.3 and as a 'tweak' if it has a leader similarity over 0.7, the percentage of entries that class as leaps is 25.9%, while the percentage of entries that class as tweaks is 64.6%. This results in a tweak over leap proportion of 2.58. If, however, we choose to be conservative and draw the line at similarity 0.5 to split entries into tweaks and leaps, the percentages of tweaks and leaps become 70% and 30%, respectively.

Contest Information

Name	Date	Number of players	Number of entries	Number of passed entries	Total number of lines
**Ants**	May 2005	167	2206	1972	18497
**Binpack**	Dec 1998	138	1455	877	6666
**Blackbox**	Nov 2006	170	6367	4600	85754
**Blockbuster**	Apr 2006	183	5922	5150	26546
**Color Bridge**	Nov 2009	117	2837	2270	14684
**Crossword**	Apr 2011	94	2280	1847	18362
**Gene Splicing**	Nov 2007	136	3285	2687	38760
**Gerrymandering**	Apr 2004	169	2392	2038	32749
**Mars Surveyor**	Jun 1999	63	1647	1371	10321
**Mastermind**	Sep 2001	123	1138	511	6322
**Molecule**	May 2002	154	1631	977	8286
**Moving Furniture**	Nov 2004	109	1834	1270	14282
**Peg Solitaire**	May 2007	119	3914	3426	19428
**Protein Folding**	Nov 2002	202	2437	1881	11901
**Sailing Home**	Nov 2010	98	3616	3175	17183
**Sensor**	Apr 2010	182	4814	4232	21503
**Sudoku**	Nov 2005	186	3061	2439	22778
**Tracking Freight**	Apr 2003	129	1661	1363	7369
**Wiring**	Apr 2008	106	4166	3707	92181

Supplementary Table 1
Dates and sample size information for all 19 contests included in the dataset

Example problem: Peg Solitaire, May 2007

This contest is based on a simple peg jumping game. In a typical game of Peg Solitaire, the board contains pegs (sometimes marbles) and at least one empty space. Pegs can be removed by jumping over them with another peg, and the aim is to remove as many pegs with a combination of jumping moves.
This implements an extended version of the original Peg Solitaire game, in which the pegs carry points, and the goal is to jump pegs in order to make the score as low as possible. This may mean it is not necessary to remove all the pegs. Each peg has a value, or weight. A move consists of one peg jumping over and thereby removing another peg. A "jump" is a horizontal or vertical move in which one peg passes over exactly one other peg and comes to rest on an empty space. Diagonal jumps are not permitted. There is a reward for every peg removed from the board according to its weight, and a penalty for each jump according to the weight of the jumping peg. The score is therefore the

difference between the value of the peg being jumped over and the jumping peg. Therefore a good score can be achieved by jumping with a low value peg over a high value peg; the bigger the difference between the values of the two pegs, the better the score. If, however, a high value peg is used to jump over a low value peg, the score decreases.

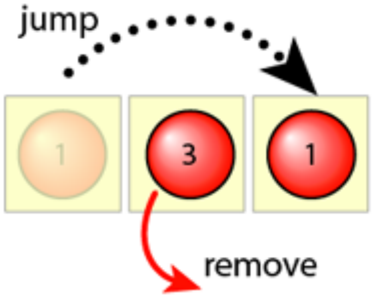

A peg with weight 1 jumps over a peg with weight 3. The reward is 3 and the penalty is 1, therefore the value of the move is 2. If several jumps in a row can be performed using the same peg, the penalty only has to be paid once.

In more detail:

- Each peg has a weight, which is always positive.
- The board is a matrix. Each positive number indicates a peg, zeros indicate empty squares, and negative numbers indicate off limits squares outside of the board.
- Every move is a four-element row vector with the format [from_row from_column to_row to_column]
- The code must return a four-column move matrix in which each row represents one move. This matrix can have any number of rows between 0 and (numpegs – 1). Any number of rows exceeding this are ignored.
- The value of each move is the sum of the removal bonus and the jumping penalty.
- Consecutive moves by a single peg only incur one jumping penalty.
- The score starts at a high value (the sum of all the peg weights). After each move, the point value of that move is subtracted from the score. The goal is to minimize the score.
- An invalid move does not generate an error – the board remains unchanged and the jumping penalty is still paid.

The overall score of an entry is a combination of three factors:

- result – the average score across all game boards
- runtime – how fast the code runs
- complexity – cyclomatic complexity - a measure of the number of independent paths through a program's source code. Typically, as this number gets higher, the program becomes less transparent and more difficult to understand.

The final score is calculated according to the equation:

$$score = k_1 * result + k_2 * e^{k_3 * runtime} + k_4 * \max(complexity - 10, 0)$$

The goal is to minimize all three factors. The lowest overall score at the end of the contest wins. An entry is disqualified if it has a runtime over 180 seconds.
